# Pregnancy outcomes in women with cesarean section scar defects and secondary infertility after vaginal repair: a multicenter observational cohort analysis

**DOI:** 10.3389/fmed.2025.1693754

**Published:** 2026-01-15

**Authors:** Yujia Yin, Yizhi Wang, Huihui Chen, Xipeng Wang

**Affiliations:** Department of Obstetrics and Gynecology, Xinhua Hospital Affiliated to Shanghai Jiao Tong University School of Medicine, Shanghai, China

**Keywords:** cesarean section scar defects, *in vitro* fertilization, pregnancy, secondary infertility, vaginal repair

## Abstract

**Background:**

Secondary infertility following cesarean section is associated with the presence of cesarean section scar defects (CSD). Vaginal repair (VR) is an effective reconstructive surgery for excising CSD, with previous evidence confirming its ability to alleviate menstrual symptoms. To study the pregnancy outcomes of CSD women after VR, we conducted an observational cohort study and analyzed the relationship between clinical characteristics and pregnancy outcomes.

**Methods:**

495 women desiring fertility among 1,085 women with CSD who underwent VR was enrolled between June 2014 and December 2021 in Xinhua Hospital and Shanghai First Maternity & Infant Hospital. All participants had undergone VR and were followed up postoperatively to document menstrual patterns and assess CSD imaging parameters. Additionally, patients’ intentions to conceive post-surgery were recorded, together with follow-up data on pregnancy outcomes.

**Results:**

In 495 CSD women desiring fertility, 44.04% achieved subsequent pregnancies following VR. The outcomes included a live birth rate of 75.43%, a miscarriage rate of 18.10%, and no instances of uterine rupture. 58.87% of CSD patients with prior secondary infertility achieved pregnancy. Patients with prior secondary infertility who achieved pregnancy exhibited a higher proportion of optimal healing postoperatively compared to those without pregnancy. Among pregnant women with prior infertility, 78.31% conceived naturally, while 21.69% resorted to *in vitro* fertilization (IVF). Notably, a higher percentage of the spontaneously pregnancy (SP) group experienced a menstrual duration of less than 7 days and demonstrated the residual myometrium thickness (TRM) exceeding 5.39 mm post-VR.

**Conclusion:**

VR surgery might improve fertility and increase IVF efficacy in CSD patients with secondary infertility. Reaching optimal healing after VR may increase the probability of successful pregnancy and natural conception.

## Introduction

Cesarean section (CS) is associated with various long-term complications, including the persistence of an unhealed uterine myometrial defect, commonly termed a uterine niche or cesarean section scar defects (CSD) (1). This condition affects 50 to 84% of women with a history of CS (2).

Approximately 30 to 40% of women with CSD exhibit postmenstrual spotting, dysmenorrhea, and chronic pelvic pain, affecting their quality of life (3, 4). Studies have established a significant correlation between the dimensions of CSD, such as the depth of the niche and the residual myometrial thickness (TRM), and the incidence of secondary infertility, defined as the inability to conceive over 12 months of unprotected intercourse despite a previous pregnancy (5–7). Furthermore, CSD adversely impact the fertility outcomes of women undergoing assisted reproductive technology (ART) (8). The accumulation of mucus or blood within niches leads to a chronic inflammatory microenvironment. This condition can diminish endometrial receptivity and obstruct embryo implantation, ultimately contributing to infertility and ART failure (9, 10). Consequently, significant improvements in fertility might be achieved through surgical repair of the residual niche.

Currently, there are three main surgical approaches for niche repair: hysteroscopy, laparoscopy, and vaginal repair surgery (VR). Studies involving laparoscopic and hysteroscopic repair have widely reported subsequent pregnancy rates ranging from 20 to 80% (11). Nonetheless, evidence regarding the effects of VR on subsequent pregnancy outcomes is limited. Additionally, reports on the treatment efficacy for patients with CSD-associated secondary infertility and the impact on *in vitro* fertilization (IVF) interventions remain scarce.

Since 2013, our team has been performing VR in treatment of CSD more than 1,200 cases. Our prior research has reported that VR can significantly alleviate uterine bleeding symptoms in patients with CSD (12, 13). Regarding pregnancy outcomes, we hypothesized that VR could enhance fertility in women with CSD pursuing subsequent pregnancies. To explore this, we conducted a multicenter ambispective observational cohort study, incorporating both retrospective data from previously diagnosed CSD patients and prospective follow-up data on postoperative outcomes, as well as clinical data from newly enrolled participants, among CSD patients who attempted pregnancy to assess the impact of VR on their pregnancy outcomes, particularly focusing on those with prior secondary infertility. This ambispective design incorporates both retrospective data from previously diagnosed CSD patients and prospective follow-up data on postoperative outcomes, as well as clinical data from newly enrolled participants.

## Methods

### Patients

**Figure 1:**
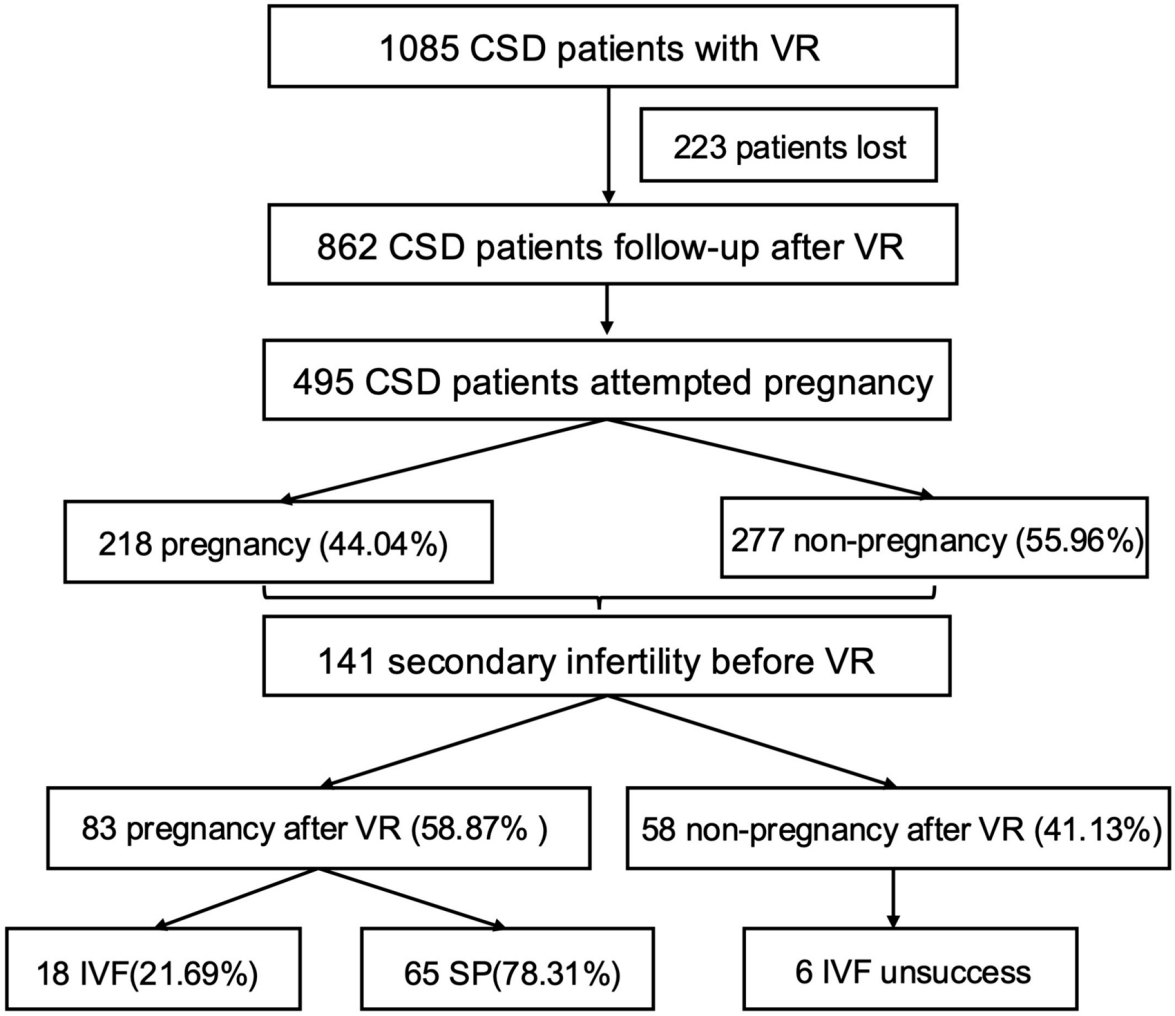
Flowchart of patients included in this study.

A total of 1,085 patients who underwent VR for CSD between June 2014 and May 2017 in Shanghai First Maternity and Infant Hospital (*n* = 501) and from May 2017 to December 2021 in Xinhua Hospital (*n* = 584) were initially enrolled. 109 patients were retrospectively analyzed based on standardized clinical records collected before the initiation of this study. The remaining patients were enrolled and followed prospectively with predefined assessment schedules and data collection procedures. Eligibility criteria included a history of one or more cesarean sections, desire for subsequent pregnancy, clinical symptoms of prolonged menstruation period or intermenstrual bleeding followed by cesarean section, and the presence of niche in the anterior lower uterine segment confirmed by transvaginal ultrasonography (TVU), shown as our prior research (14, 15). Once a niche was detected by TVU, hysteroscopy was employed for precise observation. A uterine niche was defined as a defect deeper than 2 mm on TVU or visible on hysteroscopy (8). Exclusion criteria encompassed a history of endocrine disorders, pre-cesarean menstrual irregularities, coagulation disorders, use of intrauterine devices, or the presence of any other uterine pathology, such as submucosal fibroids, endometrial polyps, or hyperplasia or carcinoma. Among 1,085 patients who underwent VR for CSD, 223 were lost to follow-up. Finally, 495 patients who attempted pregnancy were involved in the final analysis, described as Figure 1. Secondary infertility, defined as the inability to conceive over 12 months of unprotected intercourse despite a previous pregnancy, was assessed before VR (5). Only women who had previously achieved pregnancy with the same partner and demonstrated no evidence of male- or ovulatory-factor infertility were considered eligible for VR. Subsequent pregnancy confirmation was based on ultrasonographic detection of an intrauterine gestational sac. According to the method of achieving pregnancy, participants were divided into two groups: those who achieved spontaneous pregnancy (SP) and those who underwent IVF.

### Vaginal repair surgery

Surgical techniques previously detailed in our publication are concisely summarized herein (15). An initial anterior incision was made from the 3 to 9 o’clock positions, located 0.5 cm below the reflexed vesicocervical junction. Upon entering the abdominal cavity, the precise location of the uterine defect was identified. The extent of the niche in the lower uterine segment was assessed using the surgeon’s index finger in combination with an intrauterine sound. Subsequently, CSD tissue was excised, and incision margins were refined to viable myometrium using dissecting scissors before closure. All participants treated with VR by the same gynecologist and his team.

### Patient follow-up

Patients were required to adhere to a follow-up interval of 6 months post-VR to document menstrual patterns, assess CSD dimensions, and evaluate fertility desires. Menstrual data were recorded in diaries; however, approximately 10% of the participants relied on recall to report their menstrual conditions. Collected data included menstrual duration and CSD anatomical metrics such as width, depth, length, and the TRM as determined by TVU. TRM was defined as the minimal distance from the endometrium to the serosal surface across the cesarean scar in the sagittal plane. The criteria for optimal healing established in this study included a menstrual duration not exceeding 7 days and a TRM of at least 5.39 mm post-VR, which were identified in our prior research as critical prognostic factors for repair success (14). Additional baseline data compiled included CS and VR history. For patients desiring fertility, specialist gynecologists recommended a waiting period of 9 months post-VR before attempting conception, initially trying natural conception for 6 months. Long-term follow-up was subsequently required to clearly ascertain pregnancy outcomes through periodic telephone. Data were also collected and recorded on the preconception period, mode of pregnancy, gestational age, pregnancy complications, delivery mode, interval between repair and conception, and the neonatal birth weight of subsequent pregnancies. The median follow-up duration was 67.05 months.

### Statistical analysis

Continuous variables were presented as mean ± standard deviation (SD) or median with interquartile ranges, whereas categorical variables were presented as frequencies and percentages. The selection between the independent *t*-test and nonparametric tests for continuous data depended on the distribution. Categorical variables were analyzed using Pearson’s Chi-Squared test or Fisher’s exact test as appropriate. Statistical analyses were conducted utilizing SPSS software (version 25, IBM Corporation, Armonk, N.Y., United States) with a *p*-value <0.05 denoting statistical significance. The relative risk plot was generated using R software (v.4.2.2) through Hiplot Pro.^1^

## Results

### Clinical characteristics of CSD patients attempted pregnancy after VR

Among 1,085 patients who underwent VR for CSD, 223 were lost to follow-up. Finally, 495 patients who attempted pregnancy were involved in the final analysis, described as Figure 1. Among 495 CSD patients who attempted subsequent pregnancy following VR, 218 achieved pregnancies, resulting in a success rate of 44.04%. Prior to VR, 28.48% (141/495) of these patients experienced secondary infertility. Post-VR, 58.87% (83/141) of CSD patients combined with secondary infertility achieved pregnancy. Concerning gynecological outcomes in 495 CSD patients, average menstrual duration was reported as 6.03 ± 1.23 days before CS, extended to 12.96 ± 3.97 days post-cesarean, and then moderated to 7.69 ± 2.15 days after VR. It demonstrated an average TRM in TVU of 2.62 ± 1.14 mm following CS, which increased to an average of 7.18 ± 2.34 mm post-VR. Detailed baseline characteristics and gynecological symptoms before and after VR for these 495 patients desiring fertility are presented in Supplementary Table 1.

In the obstetrical outcomes for 218 pregnant patients, a total of 232 pregnancies were documented. The outcomes of these pregnancies included a 75.43% (175/232) live birth rate, a 18.10% (42/232) miscarriage rate, and notably low rates (0.43%) of scar (1/232) and tubal (1/232) pregnancies. Twenty-two individuals experienced obstetric complications, including placenta previa in 6.86% (12/175), placenta accreta spectrum in 1.14% (2/175), and postpartum hemorrhage in 4.57% (8/175), with no cases of uterine rupture or hysterectomy. Additional detailed obstetrical outcomes are shown in Table 1.

**Table 1:** Obstetrical outcomes of CSD patients with fertility desire after VR.

Characteristics	Value	No. of patients
Pregnant patients	218 (44.04%)	495
Secondary infertility before VR	141 (28.48%)	495
Pregnant patients with prior infertility	83 (58.87%)	141
Interval from VR to pregnancy (months)	20.55 ± 13.12 (1–72)	218
Pregnancy number	232 (100%)	232
1	205 (94.04%)	218
≥2	13 (5.96%)	
Pregnancy mode		
IVF	22 (9.48%)	232
SP	210 (90.52%)	
Pregnancy mode in secondary infertility		
IVF	18 (21.69%)	83
SP	65 (78.31%)	
Pregnancy outcomes		
Miscarriage	42 (18.10%)	232
Tubal pregnancy	1 (0.43%)	
Cesarean scar pregnancy	1 (0.43%)	
Ongoing pregnancy	13 (5.60%)	
Live birth	175 (75.43%)	
Pregnancy outcomes in ≥2 pregnancy		
≥2 Miscarriage	4 (30.77%)	13
≥2 Live birth	4 (30.77%)	
Delivery mode		
Cesarean section	162 (92.57%)	175
Transvaginal delivery	13 (7.43%)	
Term birth	133 (76.00%)	175
Preterm birth	42 (24.00%)	
Obstetric complication		
Placenta previa	12 (6.86%)	175
Placenta accreta spectrum	2 (1.14%)	
Postpartum hemorrhage	8 (4.57%)	
Uterine rupture	0 (0.00%)	
Hysterectomy	0 (0.00%)	
Median gestational age (weeks)	37 (4)	175
Average weight of per infant (g)	3140.8 ± 545.8 (1070–4,300)	157

Values are mean ± standard deviation (range), *n* (%), or median (interquartile range). VR, transvaginal repair; IVF, *in vitro* fertilization; SP, spontaneous pregnancy.

### Impact of clinical characteristics on reproductive outcomes in CSD patients with prior infertility

The comparison of baseline and gynecological characteristics between CSD patients with prior infertility who achieved pregnancy and those who did not showed no significant differences before VR surgery, as presented in Table 2. However, analysis of factors influencing VR surgery outcomes revealed significant associations with shorter postoperative menstrual durations (7.22 ± 1.98 vs. 8.61 ± 2.69, *p* = 0.006) and thicker TRM (7.26 ± 2.33 vs. 6.03 ± 2.08, *p* = 0.010) among patients with prior infertility who successfully conceived. Furthermore, a higher proportion of these patients achieved optimal healing postoperatively (64.81% vs. 28.95%, *p* = 0.001). Prior to VR, 21.69% (18/83) of the patients who eventually achieved pregnancy had unsuccessfully attempted IVF, compared to 12.07% (7/58) unsuccess in the non-pregnant group, with no significant differences in the number of attempts or average IVF cycles.

**Table 2:** Comparison of clinical characteristics between non-pregnancy and pregnancy in CSD patients with prior secondary infertility.

Characteristics	Non-pregnancy (*n* = 58)	Pregnancy (*n* = 83)	*p* value
*Baseline characteristics*			
Age of first CS (years)	25.78 ± 2.73 (21–33)	25.05 ± 3.04 (17–31)	0.147
Gravidity	1.84 ± 1.18 (1–6)	2.22 ± 1.29 (1–6)	0.063
Parity	1.05 ± 0.22 (1–2)	1.10 ± 0.40 (0–2)	0.406
Number of CS			
1	53 (91.38%)	72 (86.75%)	0.393
≥2	5 (8.62%)	11 (13.25%)	
Timing of CS			
Emergency	21 (36.21%)	33 (39.76%)	0.669
Elective	37 (63.79%)	50 (60.24%)	
Uterine position			
Anteflexed	26 (44.83%)	35 (42.17%)	0.754
Retroflexed	32 (55.17%)	48 (57.83%)	
Average weight of per infant (g)	3305.09 ± 371.49 (3000–4,400)	3233.33 ± 462.92 (2500–4,500)	0.269
CS to VR interval (years)	6.84 ± 3.56 (1–19)	6.98 ± 3.12 (1–19)	0.817
Age of VR (years)	32.86 ± 3.73 (24–40)	32.69 ± 4.15 (23–43)	0.797
*Gynecological characteristics*			
Duration of menstruation (days)			
Before CS	5.95 ± 1.61 (3–9)	5.83 ± 1.28 (3–9)	0.660
After CS	12.44 ± 3.07 (5–20)	11.63 ± 3.68 (4–22)	0.308
After VR	8.61 ± 2.69 (5–16)	7.22 ± 1.98 (3–14)	0.006*
TRM in TVU findings (mm)			
After CS	2.48 ± 0.91 (1–6)	2.69 ± 1.22 (1–6)	0.593
After VR	6.03 ± 2.08 (2.1–10)	7.26 ± 2.33 (2.2–13)	0.010*
Duration of menstruation after VR			
≤7 days	20 (43.48%)	41 (69.49%)	0.007*
>7 days	26 (56.52%)	18 (30.51%)	
TRM in TVU after VR			
<5.39 mm	14 (33.33%)	10 (18.18%)	0.087
≥5.39 mm	28 (66.67%)	45 (81.82%)	
Healing level after VR			
Optimal	11 (28.95%)	35 (64.81%)	0.001*
Suboptimal	27 (71.05%)	19 (35.19%)	
*Reproductive characteristics*			
IVF before VR	7 (12.07%)	18 (21.69%)	0.141
Average cycles of IVF before VR	1.71 ± 1.11 (1–4)	1.72 ± 0.90 (1–4)	0.816
IVF after VR	6 (10.34%)	18 (21.69%)	0.078
Average cycles of IVF after VR	1.67 ± 0.82 (1–3)	1.61 ± 1.04 (1–4)	0.616

Values are mean ± standard deviation (range), *n* (%), or median (interquartile range). * Means *p* < 0.05. CS, cesarean section; VR, transvaginal repair; IVF, *in vitro* fertilization; TRM, the thickness of the residual myometrium; TVU, transvaginal ultrasonography. Definition: The optimal healing criteria was a menstrual duration not exceeding 7 days and a TRM of at least 5.39 mm at 6 months following VR. An IVF cycle, a series of medical procedures that assist with conception by retrieving eggs from a woman’s ovaries, fertilizing them with sperm in a laboratory, and transferring the resulting embryos to the uterus.

### Comparison of reproductive and obstetrical outcomes by pregnancy mode in CSD patients with secondary infertility

Among the 83 pregnant patients with CSD and prior secondary infertility, 78.31% (65/83) conceived naturally (categorized as the SP group), while 21.69% (18/83) achieved pregnancy through IVF (categorized as the IVF group). Among those with unsuccessful prior IVF attempts, 66.67% (12/18) conceived naturally postoperatively. The women in the SP group underwent fewer IVF cycles prior to VR (1.42 ± 0.67) compared to those in the IVF group (2.33 ± 1.03, *p* = 0.031). Post-VR, the SP group experienced a significantly shorter time to conception (5.53 ± 5.92 months) compared to the IVF group (11.88 ± 11.03 months, *p* = 0.035). No significant differences were observed in obstetrical outcomes between the two groups postoperatively, as detailed in Table 3.

**Table 3:** Comparison of reproductive and obstetrical outcomes by pregnancy mode in CSD patients with prior secondary infertility.

Characteristics	IVF (*n* = 18)	SP (*n* = 65)	*p* value
*Reproductive characteristics*			
Preconception period before VR (months)	44.80 ± 25.03 (12–96)	32.83 ± 21.63 (12–120)	0.066
Preconception period after VR (months)	11.88 ± 11.03 (1–36)	5.53 ± 5.92 (0–24)	0.035*
Interval from VR to pregnancy (months)	23.28 ± 14.86 (10–60)	18.20 ± 12.34 (3–59)	0.125
IVF before VR	6 (33.33%)	12 (18.46%)	0.302
Average cycles of IVF before VR	2.33 ± 1.03 (1–4)	1.42 ± 0.67 (1–3)	0.031*
Average cycles of IVF after VR	1.61 ± 1.04 (1–4)	NA	NA
*Obstetrical outcomes*			
Pregnancy number			
1	17 (94.44%)	56 (86.15%)	0.584
≥2	1 (5.56%)	9 (13.85%)	
Pregnancy outcomes			
Miscarriage	4 (21.05%)	17 (22.97%)	0.897
Ongoing pregnancy	2 (10.53%)	5 (6.76%)	0.946
Live birth	13 (68.42%)	52 (70.27%)	0.875
Delivery mode			
Cesarean section	13 (100.00%)	50 (96.15%)	0.857
Transvaginal delivery	0 (0.00%)	2 (3.85%)	
Term birth	11 (84.62%)	40 (76.92%)	0.821
Preterm birth	2 (15.38%)	12 (23.08%)	
Obstetric complication			
Placenta previa	0 (0.00%)	2 (3.85%)	0.857
Placenta accreta spectrum	0 (0.00%)	1 (1.925%)	0.450
Postpartum hemorrhage	1 (7.69%)	0 (0.00%)	0.450
Median gestational age (weeks)	38 (3)	37 (3.25)	0.746
Average weight of per infant (g)	3248.46 ± 479.84 (2500–4,000)	3235.69 ± 533.19 (1070–4,300)	0.900

Values are mean ± standard deviation (range), *n* (%), or median (interquartile range). * Means *p* < 0.05. VR, transvaginal repair; IVF, *in vitro* fertilization; SP, spontaneous pregnancy. Definitions: Preconception period before VR, the interval during which patients prepare for pregnancy prior VR surgery. Preconception period after VR, the interval following VR during which patients prepare for pregnancy until they achieve successful conception. An IVF cycle, a series of medical procedures that assist with conception by retrieving eggs from a woman’s ovaries, fertilizing them with sperm in a laboratory, and transferring the resulting embryos to the uterus.

### Association between optimal healing and subsequent pregnancy mode after VR

**Figure 2:**
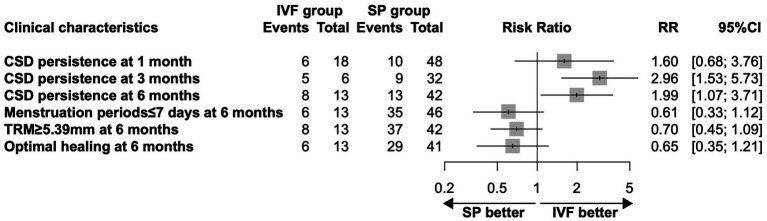
Relative risk of clinical characteristics after VR surgery with IVF vs SP. The optimal healing criteria was a menstrual duration not exceeding 7 days and a TRM of at least 5.39 mm at 6 months following VR.

Baseline and gynecological characteristics between the SP group and the IVF group are detailed in Table 4. No significant differences were observed in CS history, gynecological clinical symptoms, and TRM before VR between the SP and IVF groups. Data indicate that individuals in the SP group experienced significantly shorter menstrual durations post-VR (6.91 ± 1.72 months vs. 8.31 ± 2.46 months, *p* = 0.023) and had thicker TRM (7.60 ± 2.38 mm vs. 6.15 ± 1.82 mm, *p* = 0.035) compared to those in the IVF group. An evaluation of optimal healing post-VR showed that a higher proportion of individuals in the SP group had menstrual durations of less than 7 days (76.09%) and TRM exceeding 5.39 mm (88.10%), compared to the IVF group, where 46.15% reported menstrual durations≤7 days (*p* = 0.038) and 61.54% demonstrated TRM ≥ 5.39 mm (*p* = 0.030). Furthermore, 70.73% of patients in the SP group met the criteria for optimal healing after VR, a rate higher than that observed in the IVF group (46.15%); however, this difference was not statistically significant (*p* = 0.106). Figure 2 illustrates the relative risk ratios for clinical characteristics concerning the persistence of CSD and optimal healing post-VR, comparing IVF versus SP outcomes.

**Table 4:** Comparison of clinical characteristics by pregnancy mode in CSD patients with prior secondary infertility.

Characteristics	IVF (*n* = 18)	SP (*n* = 65)	*p* value
*Baseline characteristics*			
Age of first CS (years)	25.28 ± 3.10 (17–30)	24.98 ± 3.05 (19–31)	0.720
Gravidity	1.78 ± 1.22 (1–4)	2.34 ± 1.29 (1–6)	0.061
Parity	1.06 ± 0.42 (0–2)	1.11 ± 0.40 (0–2)	0.634
Number of CS			
1	16 (88.89%)	56 (86.15%)	0.762
≥2	2 (11.11%)	9 (13.85%)	
Timing of CS			
Emergency	6 (33.33%)	27 (41.54%)	0.529
Elective	12 (66.67%)	38 (58.46%)	
Uterine position			
Anteflexed	4 (22.22%)	31 (47.69%)	0.053
Retroflexed	14 (77.78%)	34 (52.31%)	
Average weight of per infant (g)	3283.33 ± 532.68 (2800–4,500)	3221.79 ± 452.30 (2500–4,300)	0.873
CS to VR interval (years)	7.44 ± 3.62 (4–19)	6.89 ± 3.04 (1–15)	0.664
Age of VR (years)	33.39 ± 4.31 (29–43)	32.49 ± 4.12 (23–43)	0.421
*Gynecological characteristics*			
Duration of menstruation (days)			
Before CS	5.50 ± 1.15 (4–7)	5.92 ± 1.30 (3–9)	0.164
After CS	10.59 ± 3.14 (6–15)	11.93 ± 3.80 (4–22)	0.138
After VR	8.31 ± 2.46 (5–14)	6.91 ± 1.72 (3–12)	0.023*
TRM in TVU findings (mm)			
After CS	2.92 ± 1.35 (1.3–6)	2.62 ± 1.19 (1–6)	0.435
After VR	6.15 ± 1.82 (3–10)	7.60 ± 2.38 (2.2–13)	0.035*
Duration of menstruation after VR			
≤7 days	6 (46.15%)	35 (76.09%)	0.038*
>7 days	7 (53.85%)	11 (23.91%)	
TRM in TVU after VR			
<5.39 mm	5 (38.46%)	5 (11.90%)	0.030*
≥5.39 mm	8 (61.54%)	37 (88.10%)	
Healing level after VR			
Optimal	6 (46.15%)	29 (70.73%)	0.106
Suboptimal	7 (53.85%)	12 (29.27%)	

Values are mean ± standard deviation (range), *n* (%), or median (interquartile range). * Means *p* < 0.05. CS, cesarean section; VR, transvaginal repair; IVF, *in vitro* fertilization; SP, spontaneous pregnancy; TRM, the thickness of the residual myometrium; TVU, transvaginal ultrasonography. Definition: The optimal healing criteria was a menstrual duration not exceeding 7 days and a TRM of at least 5.39 mm at 6 months following VR.

## Discussion

We observed that 44.04% of women with CSD and 58.87% of women with CSD and prior secondary infertility achieved subsequent pregnancies following VR surgery. Among these CSD patients with secondary infertility, those who achieved pregnancy were more likely to have shorter menstrual durations and thicker TRM, and to reach optimal healing postoperatively compared to those who did not conceive. Additionally, a higher proportion of women with secondary infertility who achieved natural conception met the optimal healing criteria post-VR, primarily with TRM exceeding 5.39 mm, compared to those in the IVF group. These findings suggest that VR surgery may enhance the reproductive potential of patients with CSD and secondary infertility.

Many studies have highlighted the negative impact of CSD on fertility. While ART offers potential benefits for infertile women with CSD, evidence indicates that the pregnancy rate following ART for such women is lower compared to those undergoing surgical intervention (16). Additionally, research has shown that patients with CSD diminished rates of implantation, clinical pregnancy, and live birth in the ART treatments (8). Therefore, to restore the reproductive capabilities of CSD patients, surgical repair of the CSD is recommended.

Surgical treatments such as hysteroscopic repair, laparoscopic repair, and vaginal repair have been employed to address CSD-related symptom. Regarding subsequent pregnancy rates, studies have shown a broad range: 25 to 83% after laparoscopic repair, 30 to 78% after hysteroscopic repair, and 33 to 70% after vaginal repair (11, 17–22). However, previous studies have been limited by small sample sizes and short follow-up periods, with the largest cohort of patients attempted subsequent pregnancy after repair numbering only up to 124 (23, 24), resulting in considerable variability in reported pregnancy rates across studies. As the largest study to date on VR in patients with CSD attempted subsequent pregnancy, our findings of a 44.04% pregnancy rate, 18.10% miscarriage rate, and 75.43% live birth rate offer compelling evidence. Consistent with other surgical approaches, there have been no reports of uterine rupture in pregnancies following our repair procedures.

Additionally, few studies have reported on the benefits of CSD repair surgery in enhancing success rates for patients with previous ART failures. A recent prospective study on hysteroscopic and vaginal repair for CSD indicated that 60% (12/20) of patients with prior ART failures achieved pregnancy post-surgery (25). In our study, 18 patients with secondary infertility and prior ART failures successfully conceived, with 66.67% (12/18) achieving natural conception after repair. We also observed a trend toward reduced IVF cycles after surgery, from 2.33 ± 1.03 to 1.61 ± 1.04. Compared with the published IVF cohort of women without repair surgery, which reported a miscarriage rate of 34.62% and a live birth rate of 18.99%, our post-repair IVF group demonstrated lower miscarriage rate (21.05%) and higher live birth rate (68.42%) (8). These findings suggest that VR surgery may improve ART treatment outcomes.

In our previous study, optimal healing was defined as achieving a menstrual duration of no more than 7 days and a TRM of at least 5.39 mm following vaginal repair (14). Our current study uniquely explores the relationship between VR outcomes and both reproductive outcomes and different modes of pregnancy. We found that patients achieving optimal healing post-VR were more likely to conceive successfully and naturally. This suggests that clinicians could encourage natural conception for CSD patients who meet the optimal healing criteria. For those not achieving optimal healing, additional interventions may be necessary to mitigate the impact of the uterine niche on fertility and to evaluate other factors influencing wound healing and pregnancy.

The presence of intrauterine fluid in CSD, resulting from the accumulation of mucus or blood within the defect, may hinder sperm penetration, impair endometrial receptivity, and obstruct embryo implantation, contributing to infertility (9, 26, 27). Furthermore, studies have shown elevated uterine cavity levels of inflammatory cytokines in women with CSD, linking chronic inflammation within CSD sites to subsequent infertility (10, 28). VR surgery may eliminate inflammatory cavities, reduce the residue of inflammatory effusion, thereby improving the uterine microenvironment conducive to embryo implantation and survival. Future studies incorporating pre-repair assessments of intrauterine fluid and inflammatory marker expression may yield important insights into the pathogenesis of CSD and the mechanism of surgical repair interventions.

The relatively large sample size and extended follow-up period in our study contribute to a more comprehensive understanding of pregnancy outcomes following VR surgery in women with CSD. This research also addresses the clinical relevance of reconstructive surgery for improving pregnancy and ART outcomes in CSD patients with secondary infertility. However, our study is limited by the lack of prospective design with the expective-treatment control and the absence of large-scale comparative analyses across different surgical approaches, which may introduce potential sampling bias and restrict the generalizability of our findings. In addition, recall bias related to menstrual history remains a potential limitation. Future prospective studies involving well-defined cohorts of patients with CSD who opt for expective management and desire future pregnancy are warranted to further elucidate the impact of VR surgery on obstetrical outcomes and to guide evidence-based surgical decision-making.

## Conclusion

In summary, VR surgery represents a safe and minimally invasive approach for restoring fertility and augmenting the success of IVF in women with CSD. Our results suggest that achieving optimal healing following VR may enhance the likelihood of successful pregnancy and natural conception in women with CSD and prior secondary infertility. Although the observed pregnancy outcomes appear favorable, further controlled studies are needed before broader clinical recommendations can be made.

## Data Availability

The raw data supporting the conclusions of this article will be made available by the authors, without undue reservation.

## Footnotes

1
https://hiplot.com.cn/
