# Supplementary material for: Pregnancy outcomes in women with cesarean section scar defects and secondary infertility after vaginal repair: a multicenter observational cohort analysis
Source: Front Med (Lausanne). 2026 Jan 15;12:1693754. doi: 10.3389/fmed.2025.1693754 (PMC12852386; doi:10.3389/fmed.2025.1693754)
Supplement: Supplementary file 1 [file Table_1.docx]

**Supplemental table 1. Clinical characteristics of CSD patients with fertility desire.**

| **Characteristics** | **Value** |
| --- | --- |
| **Baseline characteristics** |  |
| Age of first CS (years) | 26.07±3.38（17-36） |
| Gravidity | 2.13±1.22（1-9） |
| Parity | 1.24±0.48（0-3） |
| Number of CS |  |
| 1 | 389（78.59%） |
| ≥2 | 106（21.41%） |
| Timing of CS |  |
| Emergency | 158（40.00%） |
| Elective | 237（60.00%） |
| Uterine position |  |
| Anteflexed | 211（42.89%） |
| Retroflexed | 281（57.11%） |
| Average weight of per infant (g) | 3248.08±418.27（2000-4500） |
| CS to VR surgery interval (years) | 5.56±3.25（0-19） |
| Age of VR surgery (years) | 32.65±3.87（23-44） |
| **Complications** |  |
| Bladder injury | 1（0.20%） |
| Hematoma | 1（0.20%） |
| Infection | 0（0.00%） |
| **Gynecological outcomes** |  |
| Duration of menstruation (days) |  |
| Before CS | 6.03±1.23（3-16） |
| After CS | 12.96±3.97（4-30） |
| After VR | 7.69±2.15（3-16） |
| CSD length in TVU findings (mm) |  |
| After CS | 8.83±3.76（1-29） |
| After VR | 4.30±3.78（0-13） |
| CSD width in TVU findings (mm) |  |
| After CS | 12.02±5.38（2-30） |
| After VR | 9.14±4.18（3-22） |
| CSD depth in TVU findings (mm) |  |
| After CS | 7.25±3.44（2-24） |
| After VR | 6.08±2.99（2-15） |
| TRM in TVU findings (mm) |  |
| After CS | 2.62±1.14（0.5-8） |
| After VR | 7.18±2.34（1-13） |

Note: Values are mean ± standard deviation (range), n (%), or median (interquartile range).

Abbreviations: CS, Cesarean section; VR, transvaginal repair; TRM, the thickness of the residual myometrium; TVU, transvaginal ultrasonography.
